# “We Can Do Better”: Developing Attitudinal Scales Relevant to LGBTQ2S+ Issues—A Primer on Best Practice Recommendations for Beginners in Scale Development

**DOI:** 10.3390/bs14070611

**Published:** 2024-07-18

**Authors:** CJ Bishop, Todd Graham Morrison

**Affiliations:** 1 Psychology Program, Grenfell Campus, Memorial University of Newfoundland and Labrador, Corner Brook, NL A2H 5G4, Canada; 2 Department of Psychology and Health Studies, University of Saskatchewan, Saskatoon, SK S7N 5A5, Canada; todd.morrison@usask.ca

**Keywords:** psychometrics, measurement, sexuality, gender, lesbian, gay, bisexual, transgender, LGBTQ2S+

## Abstract

In this primer, following best practice recommendations and drawing upon their own expertise in psychometrics, the authors provide a step-by-step guide for developing measures relevant to sexual- and gender-marginalized persons (SGMPs). To ensure that readers operate from a uniform understanding, definitions for central elements of psychometric testing (e.g., reliability and validity) are provided. Then, detailed information is given about developing and refining scale items. Strategies designed to reduce a pool of items to a manageable number are also highlighted. The authors conclude this primer by discussing various forms of validation (e.g., convergent, discriminant, and known groups). To further readers’ understanding, illustrative examples from measures designed for SGMPs are brought into focus throughout.

## 1. Preamble

This primer is somewhat unorthodox. It does not contain statistical formulas or technical language that is common within academic literature. Instead, our goal is to provide a resource; a toolkit, if you will, that is easy to understand and—most importantly—easy to implement: a primer that distills what we have learned about scale development—both individually and collectively—over many years. Here, you will find a step-by-step guide detailing the processes involved in translating an idea (“I want to measure attitudes about X”) into a straightforward set of items that quantifies said idea. We will highlight the mistakes that many practitioners in our field make. And, in shocking moments of candor, we will describe our own “psychometric sins” (and how we have repented) in the hopes that, by doing so, you will not make similar errors.

We recognize that other primers exist (e.g., [1]). However, we believe that ours is of value to those with limited expertise in scale development. Specifically, in our efforts to make this primer “user friendly”, we have eliminated discussion of competing theoretical frameworks in scale development (e.g., classical test theory “versus” item response theory); narrowed our focus to attitudinal assessment (thereby removing discussion of tests of ability and concepts relevant to these sorts of tests such as distractor efficiency analysis); and jettisoned concepts that require more advanced statistical training (e.g., measurement invariance). Finally, ref. [2] once noted that the “availability of more than one valid measure of a particular construct in [any] research area is important because it allows researchers the freedom to choose that scale whose particular idiosyncrasies (of length, reading level, content sampling, etc.) best suit their particular purposes” (p. 6). We would humbly assert that a similar rationale might be applied to primers. 

Given the focus of this thematic issue, our emphasis will be on measures examining concepts relevant to LGBTQ2S+ individuals (henceforth described as sexual- and gender-marginalized persons [SGMPs]). The importance of developing psychometrically sound scales for LGBTQ2S+ topics cannot be overstated. For example, in a systematic review of scales examining discrimination against sexual minorities, ref. [3] found that only one (the Daily Heterosexist Experiences Questionnaire (DHEQ; [4])) obtained a “perfect” score on five routine indicators of psychometric soundness: content validity, criterion-related validity, construct validity, scale score reliability, and factor structure (see below for descriptions of each). Thus, although dozens of measures—appearing in numerous peer-reviewed articles (e.g., Heterosexist, Harassment, Rejection, and Discrimination Scale [5])—were assessed, apart from the DHEQ, none were classified as representing a “gold standard”. Similar results were identified across several follow-up systematic reviews focusing on scales involving transgender [6,7], bisexual [8,9], and LBGT [10] prejudice and discrimination. Each systematic review lists many different measures created to examine the construct of interest (e.g., transnegativity), yet few were found to possess meritorious psychometric properties. Across these systematic reviews, various suboptimal practices were noted. For example, it was common for many of these scales to use items originally designed for one marginalized group, such as People of Color, and “repurpose” them for LGBTQ2S+ targets. While there may be points of intersection in the lived experiences of persons marginalized because of race, sexuality, or gender, clearly, these are not interchangeable groups. 

Before commencing with key definitions, three points are worth noting. First, marginalized groups should play a central role in the development and refinement of measures pertinent to their lived realities. For instance, if researcher A, who identifies as a cisgender lesbian woman, wishes to create a scale assessing trans women’s experiences of discrimination, it is imperative that trans women be involved in this endeavor. Ideally, researcher A would partner with one or more trans women who are able to provide “lived expertise” that could be harnessed throughout the scale development process. At a minimum, researcher A should ensure that trans women are involved in evaluating the measure’s content validity. What *must* be avoided is researchers—outside SGMP communities—blithely proceeding to create measures “relevant” to SGMPs in the absence of any meaningful consultation. Second, whether a finding achieves statistical significance depends largely on the size of one’s sample. With a smaller sample, statistical significance is more difficult to attain. Thus, we recommend that one rely on effect sizes when making determinations about the “importance” of a given result. Third, in this primer, we provide various cut-offs in terms of recommended values for Cronbach’s alpha coefficient or item-total correlations. As [11] notes, in the realm of psychometrics, *any* cut-offs that are furnished represent the *opinions* of the authors and should be treated accordingly. Blind adherence to the guidelines offered by any primer is not recommended. Instead, think of the information we provide as helpful advice that may assist you in your psychometric decision-making. 

## 2. Validity

In the natural sciences, there is often consensus about how things are defined (or operationalized). Water, for example, is an inorganic compound with the chemical formula H_2_O. If you access various online sources, the same definition will appear (i.e., the chemical formula for water will not be identified as CH_2_O, which, incidentally, is the empirical formula for formaldehyde). In the realm of psychometrics, greater variability is evident in terms of how key concepts are defined. We believe that this variance is problematic because it complicates communication between researchers. If, for example, we define “convergent validity” one way, but our colleague defines it in a different way, then we are unable to discuss this concept with our colleague in a manner that is mutually intelligible (i.e., the *same* term is being used to describe *different* ideas). Inconsistent terminology is equally worrisome because it hinders the accumulation of psychometric support for a given scale. To facilitate the communication of key concepts, we will be using the conceptual definitions provided by [12]. Following this section, we also propose operational definitions of these concepts by providing instructions on *how* to measure each psychometric property, rooted in best practices. 

Validity refers to a scale assessing what the researcher intends for it to assess. For instance, if we create a measure of positive identity for SGMPs, then we want the measure to examine that construct (i.e., positive identity) and not something (potentially) related such as self-esteem. There are several forms of validity. We start with the least consequential (i.e., face validity) and continue with the increasingly complex ones (i.e., content validity, criterion-related validity, construct validity, and factorial validity). 

### 2.1. Face Validity

This is the most basic form of validation. It involves one or more individuals reviewing scale items and telling the researcher(s) whether the items *appear* to measure what they are intended to measure. In other words, are the items face valid? It should be noted that high levels of face validity may not always be beneficial. In the cases of attitudinal measures involving SGMPs, scales with high face validity may be prone to soliciting responses affected by social desirability bias (i.e., responses intended to adhere to the expectations of others). For example, if participants deduce a measure is assessing their attitudes toward bisexual women, they may respond in ways that reflect a stance of allyship rather than their actual feelings. Given its basic nature, this form of validation is not particularly compelling.

### 2.2. Content Validity

This form of validation may be viewed as a “step up” from face validity. To determine whether a set of items are content valid, you would recruit experts in psychometrics and in the topic being measured. These experts rate each individual item on various criteria such as relevance (i.e., is each item relevant to the topic being measured?), representativeness (i.e., do the items capture the full domain of content being considered?), and clarity (i.e., is each item written in a manner that would be understandable to the average respondent?). Items that are deemed by content experts to be relevant, representative, and clear should be retained. Items that do not satisfy these characteristics should be revised or eliminated. 

### 2.3. Criterion-Related Validity

To assess this type of validity, you would test the correlation between scores on the scale you have created and scores on another psychometrically sound scale designed to assess the *same* construct. For example, if we developed a measure of old-fashioned homonegativity toward gay men (i.e., negativity rooted in traditional religious and moral objections to male homosexuality [13]), we could assess our measure’s criterion-related validity by correlating scores on our measure with scores on a gold standard indicator of the *same* concept (e.g., Attitudes toward Gay Men scale (ATG; [14])). A strong correlation would suggest that scores on our new measure evidence criterion-related validity. Of course, the stronger the correlation, the greater the conceptual overlap between our new measure and the gold standard. This, in turn, necessitates that we provide a compelling rationale for the existence of the measure we are creating. In other words, how does our new measure improve upon the measure (or measures) that exist already? 

Criterion-related validity may be partitioned into two main subtypes: concurrent and predictive. Concurrent validity involves distributing your new measure and the gold standard measure at the *same time* to a group of respondents and testing the correlation between the scales’ scores. The example provided earlier, which focuses on testing the correlation between scores on our newly created measure of old-fashioned homonegativity and the ATG [14], illustrates this form of criterion-related validity. 

Predictive validity is more complicated. Here, you would distribute your measure at Time 1 and then examine the correlation between scores on your measure and scores on an indicator/outcome variable of a related concept collected at Time 2. For example, suppose that we created a measure examining chronic stressors experienced by SGMPs. To determine whether scores on this measure evidence predictive validity, we might investigate the correlation between scores on our measure and respondents’ self-perceptions of stress collected three months later. We would expect a positive correlation between these two indicators (i.e., individuals reporting greater levels of chronic stress on our measure also should self-report greater stress levels at a subsequent point in time). Given its use of different time points and resultant concerns about respondent attrition, predictive validity is seldom tested in sexual and gender minority studies.

Based on these definitions of criterion-related validity, two obvious questions emerge: (1) What happens when a gold standard indicator does not exist? (2) What happens if there is no relevant construct that you can measure at a future point in time? Under such circumstances, criterion-related validity is not an option; instead, researchers must rely on testing construct validity. 

### 2.4. Construct Validity

With this form of validation, theory and/or past empirical work are used to guide the creation of testable predictions (i.e., hypotheses). For instance, when [13] created the Modern Homonegativity Scale (MHS), they predicted that higher scores on the MHS, which suggest greater negativity toward gay/lesbian persons, would correlate positively with greater levels of religiosity, political conservatism, and neosexism (i.e., more subtle sexist beliefs about women (see [15])). Each of these predictions was supported statistically (i.e., participants who reported greater homonegativity on the MHS also reported being more conservative, more religious, and more sexist). The support afforded these predictions offered strands of evidence attesting to the construct validity of MHS scores.

Construct validity may be divided into three types. First, we have convergent validity. This form of construct validity involves testing correlations between scores on your measure and scores on psychometrically robust measures that, for theoretical and/or empirical reasons, should be correlated with each other. The examples provided earlier for the MHS reflect convergent validation. It is important that readers understand this definition. A common experience for each of us, in our capacity as members of journal editorial boards, is for researchers to claim evidence of “concurrent validity” based on correlations between scores on their new measure and a psychometrically sound scale that is theoretically “very similar”. We must reiterate that concurrent validity can be established only when the comparative scale measures the same construct as your new scale. Therefore, despite claims that concurrent validity has been tested, these researchers have assessed convergent validity.

The second type of construct validity is known as discriminant (or divergent) validity. Here, the goal is to demonstrate—again, for theoretical and/or empirical reasons—that scores on your measure are *unrelated* or not significantly correlated with scores on another psychometrically sound scale. Discriminant validity is intended to work in tandem with convergent validity to provide additional strands of evidence that your scale measures what it is intended to measure. Discriminant validity is routinely misinterpreted. Why? Likely because of its name, which suggests it is testing whether scores on your measure correlate negatively with (i.e., diverge from) scores on another measure. Unfortunately, psychometric terminology can be counterintuitive: in the case of discriminant (or divergent) validity, non-significant or near-zero correlations are the goal, and *not* statistically significant negative correlations. 

Another critical point: discriminant (or divergent) validity cannot be tested in a theoretical/empirical vacuum. It is incumbent on *you* to furnish a rationale for the hypotheses you generate. If, for example, we correlated scores on [16]’s Positive Bisexual Identity Scale (PBI) with scores on a scale assessing concerns about air pollution, we (likely) would obtain a statistically non-significant correlation. However, as there is no theoretical and/or empirical rationale linking these two constructs, this finding would *not* constitute evidence of the PBI’s discriminant (divergent) validity. 

The third and final type of construct validity is known-group (sometimes referred to as discriminative) validity. Examining this type of validity involves comparing groups that, for theoretical and/or empirical reasons, *should* differ on the measure being created. For instance, researchers have found that cisgender heterosexual men report more negative attitudes toward gay men than do cisgender heterosexual women. Testing the known-groups validity of a new measure of homonegativity, for example, would involve comparing men and women’s scores on the new scale. If, as predicted, the score for men is statistically significantly higher than the score for women, one strand of evidence has been furnished in support of our measure’s known-group validity. Similarly, one might test whether bisexual respondents, who report being open about their sexual identity, evidence higher scores on the Positive Bisexual Identity Scale (PBI; [16]) than their closeted counterparts. If the expected group difference emerges, this finding would provide a strand of evidence in support of the known-group validity of the PBI. It is critical to note, however, that using probability values (e.g., *p* < 0.05) to denote whether a finding is statistically significant is problematic for various conceptual and mathematical reasons (see [17]). Therefore, as we note earlier, we strongly advise that researchers also provide an indicator of a finding’s practical significance such as Cohen’s *d* (see [18]).

### 2.5. Factorial Validity

This form of validity involves using statistical techniques called exploratory factor analysis (EFA) and confirmatory factor analysis (CFA) to determine whether items representing the same construct group together. For example, ref. [19] developed a scale examining nonbinary persons’ experiences with microaggressions (i.e., verbal, nonverbal, or environmental insults that communicate negative messages to members of marginalized groups). The items that were kept by the authors focused on concepts such as negation of identity; inauthenticity (i.e., the claim nonbinary identities are not “real”); deadnaming (i.e., referring to an individual by the name they used prior to transitioning); trans exclusion (i.e., rejection by trans individuals with binary identities); and misuse of gendered terminology (i.e., purposefully using identifiers that conflict with one’s nonbinary identity like “bro” or “girl”). Each of these concepts or, to be more precise, latent themes, could be regarded as a separate factor. Thus, for [19]’s measure, a five-factor solution would be expected (i.e., items concerning negation of identity would constitute one factor, items focusing on deadnaming would comprise another factor, items pertaining to misuse of gendered terminology would comprise another factor, and so on). In this example, factorial validity would be demonstrated if a five-factor solution emerged, with each item loading uniquely on its designated factor (i.e., an item about deadnaming should load on the deadnaming factor and not on another factor such as perceptions of inauthenticity). Conversely, Ref. [13]’s MHS possesses a single factor or is *unidimensional* (i.e., each item on the MHS loads on the same factor).

## 3. Reliability 

The final set of definitions that we need to provide concerns reliability. This term refers to the degree to which participants respond *consistently* across items on a measure. As mentioned earlier, ref. [19] developed a scale examining nonbinary persons’ experiences with microaggressions. Given that the items comprising this measure assess the *same* construct (i.e., personal experience with microaggressions), one would anticipate that respondents would select similar response options *across* items. For example, if someone reports “never” having experienced people telling them that it is “difficult for them to use the pronouns that I want them to use”, then this person also should be more likely to report “never” hearing people complain about “how difficult it is for them to get my [preferred pronouns] right”. In other words, participants should respond in a consistent manner across different items used to assess nonbinary microaggressions.

Multiple forms of reliability exist (e.g., alternate form); however, only two types are used commonly in sexual and gender minority studies.

### 3.1. Test–Retest

The purpose of test–retest reliability is to ensure that participants’ responses to a scale are stable over time. This form of reliability involves administering the measure of interest at Time 1 and then presenting it again to the *same* respondents two to four weeks later (Time 2). Participant attrition is a concern (i.e., respondents may drop out before Time 2). Additionally, researchers need to be cognizant of any events that might occur between Times 1 and 2, and which have the potential to influence participants’ responses to the measure of interest. For example, suppose that we assessed SGMPs’ positive self-identity at Time 1. Then, prior to completing the measure again at Time 2, a well-publicized event underscoring mainstream society’s enmity toward these groups surfaced (e.g., in 2023, Target stores removed LGBTQ2S+ merchandise due to concerns about employees’ “safety”). The coverage of that event might have implications for how participants respond when they complete the measure a second time (i.e., their positive self-identity scores may decrease). This (unanticipated) change would compromise our test–retest reliability coefficient.

### 3.2. Internal Consistency Estimate

This form of reliability, typically measured using Cronbach’s alpha coefficient, is used most frequently. The way to think about Cronbach’s alpha is to imagine the following scenario. You have a 6-item scale and wish to assess response consistency across the items. There are various ways to divide that scale into what are known as split-halves (items 1, 2, and 3 versus items 4, 5, and 6; items 1, 3, and 6 versus items 2, 4, and 5, and so on). Each correlation between the different split-halves would be unique (i.e., the correlation between the sum of items 1–3 and the sum of items 4–6 may differ slightly from the correlation between the sum of items 1, 3, and 6 and the sum of items 2, 4, and 5). Cronbach’s alpha computes *the average* of all possible split-half estimates. In the case of our hypothetical 6-item scale, 10 different combinations of split-halves exist. Cronbach’s alpha offers a single average of those combinations. Despite its popularity, readers should be aware that using Cronbach’s alpha coefficient to assess scale score reliability has been identified as problematic for various conceptual and mathematical reasons (see [20]). In response to growing concerns about Cronbach’s alpha, we are seeing more researchers use alternate indicators of reliability such as McDonald’s Omega (see [21]). The latter is available through R software and, more recently, as a macro for SPSS and SAS. We believe that the latter development will increase researchers’ use of omega) 

It is worth noting that Cronbach’s alpha provides an *estimate* of internal consistency (i.e., item interrelatedness). Recommendations of what constitutes a “good” alpha coefficient vary but typically range from a minimum value of 0.70 (e.g., [22]) to a maximum value of 0.90 (e.g., [11]). Given that some degree of flexibility should always be observed in psychometric testing, we recommend setting your desired alpha coefficient range between 0.80 and 0.92 (Please note that all recommended alpha coefficient thresholds are the opinion of their respective authors, and our recommendations should not be viewed any differently [11].). A maximum value of 0.92 will accommodate situations where coefficients meet or slightly exceed [11]’s value. We recommend a minimum value of no less than 0.80 (contrary to often cited recommendations) due to a commonly overlooked limitation of Cronbach’s alpha. Specifically, Cronbach’s alpha increases as the number of scale items increases. Thus, if your pool of items is large enough, Cronbach’s alpha will be high, even if the items themselves are unrelated. Therefore, we believe that a minimum value of 0.70 is too easy to attain when many items are being tested. Even with our more conservative recommended range of desirable Cronbach’s alpha coefficients, be aware that these values still do not guarantee that your items are strongly intercorrelated; you still may be observing a value that is attributable to scale length. Given this limitation, when using Cronbach’s alpha, we recommend that researchers adopt the expression “scale score reliability” rather than “internal consistency”. 

With Cronbach’s alpha, always keep in mind that bigger is not necessarily better. To reiterate, you want this coefficient to fall between 0.80 and 0.92. We contend that values exceeding 0.92 suggest that some items might be redundant (i.e., as alpha coefficients approach 1.0, it becomes increasingly likely that you have items that are gathering near-identical pieces of information). Redundant items are problematic because they violate the test principle of parsimony and may contribute to content under-representation. The latter means that, when multiple items assess the same segment of a given content domain, other segments may be inadvertently omitted (i.e., under-represented). To illustrate, ref. [19] reported that nonbinary microaggressions could fall into different categories such as deadnaming (i.e., referring to an individual by the name they used prior to transitioning); trans exclusion (i.e., rejection by trans individuals with binary identities); and inauthenticity (i.e., the claim that nonbinary identities are not “real”). If the researchers created a pool of 50 items and 40 of those items focused on deadnaming, content under-representation would be a problem (i.e., an insufficient number of items would focus on other types of nonbinary microaggressions).

Also, when reporting your scale score reliability coefficients, we recommend that you include 95% confidence intervals for each value. Remember that scale score reliability coefficients are estimates and confidence intervals inform the reader that the true value exists somewhere between its lower and upper bounds [23,24]. In essence, you are providing readers with the range of your estimate.

Before concluding this section on definitions, a few points are worth emphasizing. You will notice that, throughout this paper, we focus on scale scores; that is, we do not refer to measures themselves as “being valid” or as “being reliable”. The reason for this is simple: both reliability and validity are properties of *scale scores* and, thus, are unique to each sample. They are not immutable characteristics of a measure; thus, it is inaccurate to assert that scale X is “reliable and valid”. The fact that reliability and validity are score-based highlights the iterative nature of psychometric testing. Examining scale score validity is an ongoing process, one which involves disparate samples and validation coefficients. In other words, just because a measure appears “reliable and valid” with one sample does not necessarily mean it will remain “reliable and valid” when used with another sample. 

The score-based nature of reliability also necessitates that you measure Cronbach’s alpha (or McDonald’s Omega) with each sample (or subsample) that completes your scale as well as any other scales that you are using for validation purposes. As peer reviewers and members of editorial boards, we have observed many (many!) authors using previously published tests of reliability in support of their claim that measures X, Y, and Z are “reliable”. Again, just because a good scale score reliability coefficient has been obtained with one sample does not mean that it will be obtained with your sample. 

#### Extending Considerations to Include Validity

The same logic regarding the reassessment of internal consistency estimates for each new sample may be extended to validity. Therefore, when using multiple scales, we recommend that researchers provide a correlation table. Then, in the Results section, researchers should briefly outline the correlations that are obtained (both statistically significant and non-significant) and whether these map onto predicted expectations based on the relevant literature. This type of information provides strands of support for convergent and divergent validities. We also recommend that known-group comparisons be reported for all scales. These may appear as gender comparisons, which can be tested using independent sample *t*-tests. Again, if observed group differences map onto what is expected, given available published evidence, then the reader will be reassured that scale scores are valid. 

Thinking of psychometric testing as a process that unfolds continuously also illustrates that measures themselves are not static. Items that were relevant in 1985 might not remain relevant in 2024. For instance, as mentioned earlier, ref. [13] published an article detailing the construction of the MHS. The intent of this measure was to capture “modern” forms of negativity toward gay men and lesbian women. However, as the MHS is more than twenty years old, the relevance of its items (i.e., its content validity) may be questioned. In the past, heterosexual respondents might have “strongly agreed/agreed” that gay men/lesbian women “use their sexual orientation so they can obtain special privileges”. It is critical to determine if this belief (and hence the item itself) remains salient. Using out-of-date measures is not a trivial concern. Artificial floor or ceiling effects in which most people reject or endorse a set of items can lead to erroneous conclusions about broad social attitudes, which, in turn, can have negative implications for public policy. For example, consistent low scores (i.e., floor effects) on the MHS may be interpreted as evidence of gay/lesbian individuals having reached parity with sexual majority individuals. A result of this misinterpretation may be the reduction or cancelation of funding for important LGBTQ2S+ organizations rather than the acknowledgment that a measure published more than twenty years ago is anachronistic.

### 3.3. Sample Considerations 

Psychometric testing is a “big sample” exercise. Importantly, how this “big sample” manifests itself is at your discretion. You might want to use four samples of minimum 200 respondents each or one large sample of at least 800 respondents. The reason for a specific minimum sample size of 200 participants is based on best practice recommendations for different forms of psychometric testing. For example, ref. [25] recommend at least 200 participants for an EFA. Similarly, ref. [26] recommends a minimum of 200 participants for Cronbach’s alpha testing. Since various psychometric indicators will be assessed using the same sample (except where exceptions are noted), it makes sense to ensure recommended sample size thresholds for EFA and Cronbach’s alpha are adhered to and applied to all other metrics. Be warned: using a large sample of 800+ respondents is a bit more complicated logistically because you will want to create subgroups (e.g., 200 per group), with each subgroup receiving the pool of items that you have created and *different* validation measures. Using subgroups allows you to test multiple strands of evidence for validity without placing an undue burden on participants, who, after all, have volunteered to help you! It also allows you to carry out different forms of factorial validity testing (see below) without having to recruit a new sample. 

For instance, researcher A has created a measure testing the discrimination experiences of nonbinary persons. They distribute the measure to 800 self-identified nonbinary individuals and distribute 10 measures that they believe, for theoretical and/or empirical reasons, will correlate with self-reported experiences of discrimination. The resultant survey consists of 315 items (25 items created by the researcher and 290 items representing 10 different measures). As you can imagine, respondents would find completing a survey of this size rather daunting. Importantly, the data they provide might be suboptimal (i.e., they may skip questions, satisfice, etc.). In contrast, researcher B distributes their pool of items to 800 self-identified nonbinary individuals. However, they divvy up the validation measures (i.e., validation measures 1–3 are given to respondents 1, 4, 7, 10, 13, and so on; validation measures 4–6 are assigned to respondents 2, 5, 8, 11, 14, and so on; and validation measures 7–9 are distributed to respondents 3, 6, 9, 12, 15, etc. Validation measure 10 is given to all respondents). The practice of randomly allocating participants to validation measures decreases the length of the survey which, in turn, *should* lessen boredom and fatigue.

Some might be concerned that dividing a sample into smaller subsamples will introduce systematic biases (e.g., one subsample may consist of participants who are older or better educated than the other subsample[s]). This concern may be addressed by statistically comparing the subsamples on any demographic characteristics of interest. To illustrate, the age and site of residence (urban versus rural) of subsamples 1, 2, 3, and 4 are compared. No statistically significant differences emerge (i.e., the mean ages and the proportions residing in rural versus urban locations across the subsamples do not differ). Thus, as the composition of the subsamples appears to be similar, one might collapse them into a single (larger) sample. It should be noted, however, that demographic differences across subsamples are concerning only when one has reason to believe that said differences have implications for scores on the measure being developed. 

## 4. Stepwise Instructions with Additional Context 

Now that key definitions have been provided, it is time to outline the steps involved in constructing a scale. We will begin with “topic selection”—a process that can receive short shrift from those with limited psychometric training. 

### 4.1. Selecting a Construct

Selecting a construct to measure may seem like a simple task (i.e., “I want to measure X”). However, this process should involve equal measures of forethought and critical analysis. A selected construct must be sufficiently robust to warrant the investment required by psychometric testing and, yet, not be so broad that the scale scores are meaningless. Suppose that researcher A is interested in measuring a broad construct such as “discrimination experienced by transgender individuals”. Superficially, this sounds like a reasonable and important construct to examine. Greater depth of analysis, however, would suggest that this construct is so large and cumbersome that the proposed measure would be difficult to create, exceedingly long, and possibly of limited value. 

Consider that “discrimination” itself may manifest as “blatant”, “subtle”, or “covert” [27]. Within these three subtypes are activities and behaviors that may be classified as “legal” or “illegal” depending on their severity and the resultant effects on the target. To create a suitable scale, each of these different subtypes of discrimination would need to be acknowledged. Furthermore, consider that the targets of discrimination are not homogenous. The transgender population, for example, contains many different subgroups (e.g., transgender men, transgender women, and gender nonbinary). A hypothetical scale would need to ensure that items pertaining to each subgroup are included and that they can parse out key differences among subgroups. 

As a result, we recommend being specific. Recall that [19] decided to evaluate microaggressions experienced by nonbinary persons. By focusing on a specific form of subtle discrimination (i.e., microaggressions) experienced by a certain subgroup of transgender individuals (i.e., nonbinary persons), the authors were able to identify and consider subtle aspects of this construct that may have otherwise been overlooked. 

### 4.2. Generating Scale Items

Earlier, we mentioned that “bigger is not necessarily better” when it comes to Cronbach’s alpha coefficient. However, with respect to your preliminary pool of items, your personal mantra should be “bigger *is* better”. You want to create a large pool of items because many of those items will be eliminated when they are assessed by experts (i.e., the items go through the process of content validation). 

We each learned this lesson after publishing various papers. For example, TGM co-authored a paper describing the construction of a measure entitled The Anti-Fat Attitudes Scale (AFAS) [28]. In this article, he detailed concerns with the principal instrument used to measure this construct. However, what he and his co-author failed to do was specify the parameters of the construct and develop a large pool of items to represent those parameters. Instead, they started—and finished—with five items. The authors did not seek out content experts to evaluate those items, nor did they pilot test them with “lay experts” (i.e., overweight individuals) to determine if they felt items on the AFAS reflected common prejudices directed at persons perceived to be overweight. The value of the AFAS was compromised because the authors did not test its content representativeness. It is possible that the totality of anti-fat prejudice can be captured by five items; possible, but unlikely. Similarly, CJB published an article examining heterosexual participants’ homonegative reactions in response to same-sex dyad stimuli and an interactive gay male target. His goal was to control the effects of participants’ attitudes toward public displays of affection (PDAs) given the physical intimacy displayed by the dyadic targets. At the time of data collection, a relevant measure of attitudes toward PDAs was not available so one needed to be created. The author created *four* items that were neither evaluated by content experts nor pilot-tested [29]. 

It is important that the items you generate are rooted within the three pillars critical to content validity: (1) the relevant literature; (2) input from relevant stakeholders; and (3) input from content experts [30]. It is not uncommon for researchers to create items they *think* map onto their topic of interest without paying sufficient attention to the body of literature that exists about that construct. Even in situations where a researcher reviews a handful of relevant articles, there is still the risk of overlooking important aspects of the construct that may not be reflected in the subset of papers reviewed. While a systematic assessment may be an unreasonable expectation, researchers should strive to include as much of the relevant literature as possible during the item generation process.

To assist with creating scale items, we recommend that, first, you consult the relevant literature. For instance, if you wanted to develop a measure examining attitudes toward same-sex fathers, a good place to start would be reviewing research on this topic (ideally, articles published within the last five years). At this stage, both qualitative and quantitative studies should be used. For instance, suppose you read a qualitative study in which a group of gay fathers reported that others had expressed concerns about the fathers’ ability to provide both male and female role models. You might develop several questions to reflect this concern (e.g., “Gay fathers do not provide their child/children with female role models”). In your review of the literature, you also might encounter scales that, while flawed, possess a sprinkling of items that you think warrant consideration. Please note that if you wish to add these items to your pool, you must contact the author(s) in question to obtain permission to do so. 

Finally, if you are measuring a novel construct, there may be little published research to draw upon. In such cases, you must rely on more anecdotal sources of inspiration such as conversations with friends/colleagues, self-reflection about the topic, and social media discussions. 

We also recommend supplementing a review of the literature with a qualitative component. Focus groups with relevant stakeholders, for instance, can reveal additional facets of the topic that, for whatever reason, may not be reflected in the literature. Imagine that you are creating a measure of attitudes toward two-mother (e.g., lesbian) parenting. It is important to complete a deep dive into the relevant literature pertaining to this topic. However, consulting with women who parent with a same-sex partner can offer additional crucial information that may not be captured by the published research. The inclusion of content experts is discussed in greater detail below. 

During initial item development, do not agonize over wording and issues such as clarity and representativeness. Simply create your items! Later, you will have time to determine if item 27 is double-barreled or if item 5 is too wordy. Also, at this juncture, do not be concerned about content overlap across items. Let your mantra be the following: generate, generate, generate! 

Based on our experience, we recommend that scales be 10 to 20 items in length. Fewer items can lead to construct under-representation; more than 20, and respondent fatigue may become a concern. Given these endpoints (minimum 10 items; maximum 20 items), your initial pool should comprise no fewer than 50 to 100 items, denoting a conservative retention rate of 20%. Two points are worth noting here. First, as with all psychometric primers, the guidelines we provide reflect our opinions. They are not incontrovertible facts. Second, scale development is not akin to following a recipe. Thus, the proportions we offer should be viewed as rough guidelines. For instance, there may be times when a researcher generates an initial pool of 100 or more items and there may be times when 30 items is sufficient. When it comes to creating a scale, common sense should prevail rather than dogmatic adherence to advice from experts.

In the past, it was common for scale developers to generate a mixture of items that were positively and negatively keyed. For example, in [13]’s Modern Homonegativity Scale, most of the items are keyed (i.e., scored) in one direction (e.g., “Gay men should stop complaining about the way they are treated in society, and simply get on with their lives”). For these items, agreement denotes stronger endorsement of modern homonegativity. However, a few items are reverse keyed (e.g., “Gay men still need to protest for equal rights”). With these items, agreement reflects *weaker* endorsement of modern homonegativity. When you have a mixture of positively and negatively keyed items, you must ensure that they are all keyed (scored) in the same direction. If you fail to do this, the total score on your measure will be unintelligible. Additionally, you might end up with a negative Cronbach’s alpha coefficient. A negative Cronbach’s alpha coefficient tells you that something is “off” about your pool of items. Typically, the culprit is failing to rescore items that are negatively keyed. Inspecting the output entitled “corrected item-total correlation” helps to identify any items that are problematic (i.e., these items will have substantial negative correlations with the sum of all other items). The one thing researchers should not do is ignore the fact that a negative Cronbach’s alpha has been obtained. 

Using a mixture of positively and negatively keyed items was viewed as sound psychometric practice because, at the time, it was seen as a way of helping researchers identify participants that were not paying attention. Specifically, if someone selected “strongly agree” to both “Gay men should stop complaining about the way they are treated in society, and simply get on with their lives” and “Gay men still need to protest for equal rights”, this response pattern would suggest that the participant was not reading the attitude statements carefully. 

However, most psychometrists now agree that using items keyed (scored) in different directions is problematic (see, for example, ref. [31]). First, it can be difficult to create items in which strong disagreement and strong agreement reflect the same attitudinal position. Second, negatively keyed items often rely on the use of negation (e.g., “do not”, “will not”, etc.), which can be confusing to participants. Increases in confusion heighten random measurement error, which, in turn, shrinks scale score reliability and the magnitude of correlations in general. For these reasons, we recommend that all scale items be keyed in the same direction such that *higher* scores reflect *more* of the construct. 

To address the issue of careless responding, we routinely embed one to two attention checks into the pool of items being tested. For example, “For this question, please select ‘agree’ as your response”. Any respondent who does not follow this instruction (i.e., selects an option other than “agree”) would be seen as providing questionable data. Researchers then should flag those participants who failed to answer the attention check items correctly. Their data should be retained but not used when the researcher conducts further psychometric testing. 

When creating scale items, you want them to be accessible, straightforward, and to reflect a single idea (i.e., double-barreled items must be avoided). Readability software, of your choice, should be used to assess items’ accessibility and straightforwardness (i.e., aim for the reading level of an average 11- to 12-year-old). Do not rely upon your own assessment of items’ readability, nor the assessments of undergraduate/graduate students or members of your research lab/team. Always err on the side of making items simpler rather than more complex. If participants do not understand the words that appear on a scale, they may try to decipher what those words mean. If they make an erroneous guess (e.g., assume that the word “augment” means to decrease or the word “deleterious” means helpful), the value of your data has been compromised. 

Researchers should avoid using slang expressions, as these can impose temporal limitations on the value of a scale. Slang that is popular now may be incomprehensible in a few years (e.g., today, younger gay males would likely recognize the term “straight acting” while being unaware of the now anachronistic term “Castro clone”). Idioms (e.g., “bite the bullet” or “beat around the bush”) and proverbs (e.g., “birds of a feather, flock together”) also should be avoided. Finally, researchers should consider carefully whether they wish to include coarse language or pejoratives that may be deemed offensive by some respondents. Blanket condemnation of such terminology is inappropriate; in some cases, slurs or other derogatory terms may be seen as critical elements of a scale. For instance, if we wanted to assess gay men’s experiences of verbal discrimination, we may feel justified in creating an item such as “In the past 4 weeks, I have been called a ‘fag’”. However, we should not be surprised if ethics boards express reservations about the use of this type of item nor should we be surprised if (some) respondents complain about the item’s content. 

You also want to ensure that the response format you adopt “fits” with the items. If, for example, you are interested in determining how often respondents experience an event, then “strongly agree” to “strongly disagree” would be inappropriate. Instead, a “never” to “always” response format would make more sense. In addition, beware of crafting items that suggest temporality (e.g., “sometimes”, “rarely”, “usually”, etc.). Employing temporal words can result in your items being incomprehensible. Take, for example, the following item: “Sometimes, I get upset when I think about how trans people are portrayed in mainstream media”. If the response format is “never”, “rarely”, “sometimes”, “often”, and “always”, then how does a participant make an intelligible response? Selecting “often” for this item, would result in “I ‘often’ sometimes get upset about how trans people are portrayed in mainstream media”.

We recommend using a 5- or 7-point Likert-type response format (see [32]). Having too few response options may lead to insufficient variability; having too many, and participants might become confused. Remember: people tend not to think in very granular terms; consequently, they may be unable to differentiate—in a meaningful way—among options such as “slightly agree”, “agree”, and “strongly agree”. Notice, too, that we recommend using an *odd* number of response options; doing so mitigates forced choice responding in which participants are required to “possess” an attitude about your scale items. A useful rule of thumb is that Likert scales with an even number of response options likely reflect forced choice scenarios. On the other hand, Likert scales with an odd number of response options likely have a neutral position built in.

The following response format is forced choice: strongly agree, agree, disagree, and strongly disagree (i.e., four response options). In this case, participants must select an option which denotes a particular position (agreement or disagreement). However, what happens if respondents are uncertain about their attitude or have not yet formed a specific attitude in relation to the item in question? Non-attitudes may surface; that is, participants may “agree” or “disagree” with a scale item but only because they do not have the option of selecting “no opinion” or “don’t know” (see [33]). Forced choice responding also may elicit satisficing behaviors whereby participants select the first plausible response option rather than the most suitable one (i.e., “None of these items really apply to me but this option seems like it *could* reflect my belief/opinion”). To avoid non-attitudes and/or satisficing, we recommend that researchers include options such as “don’t know”, “not applicable”, or “neither agree nor disagree”. These options may appear as the midpoint or be offered as an adjacent option (i.e., their placement will depend on the scale items and the response format that is used). Respondents may be reluctant to select “no opinion” or “don’t know” as a response option because doing so may have implications for their self-esteem [33]. We recommend that researchers “normalize” this process by emphasizing its acceptability in the instructions at the start of the survey.

We do not recommend using dichotomous response formats (e.g., “yes” versus “no”, “agree” versus “disagree”, and “true” versus “false”). They are reductionistic. Also, many researchers are unaware that binary data require the use of complex statistics (Technically, Likert scales constitute an ordinal form of measurement. However, it is common practice for Likert scales to be seen as providing interval-type data (see [34]).).

When it comes to response options, clarity is key. In the courses we teach, we routinely ask students to operationalize terms such as “sometimes” or “fairly often”. We are astounded at the variability that emerges—even among individuals who are quite homogeneous (i.e., they fit the profile of “typical” university students enrolled in psychology courses). To some, “fairly often” is “3 times per week” whereas to others, “fairly often” suggests an event occurring 4 to 5 times per month. Similar variation is noted for “sometimes”, “seldom”, and “rarely”. To maximize clarity, we recommend that researchers add numeric qualifiers, where appropriate: never (0 times per week); rarely (at most, once per month); sometimes (at most, 2–3 times per month); and so on. This type of specificity ensures that respondents are operating from the same temporal perspective.

Please note that, depending on your research goals, an alternative to Likert response options may be sought. For example, a semantic differential (SD) scale would offer participants the same number of response options. The key difference between these two scale types is that SD scales are anchored using “bipolar” adjectives (e.g., “bad” to “good” or “cold” to “hot”) while Likert scales assess levels of agreement (or disagreement) with each item. SD scales may be useful in assessing participants’ evaluations of specific constructs. However, be aware that using “bipolar” adjectives may carry some limitations. Consider a 5-point semantic differential scale assessing perceptions of transgender women from “cold” to “warm”. “Cold” would constitute the first of the five options with “warm” the fifth. Concerns arise when one considers that options 2, 3, and 4 do not possess textual indicators given that they reflect points *between* the poles. Researchers are unable to determine how participants are conceptualizing the differences between each numerical option. These differences may have implications for interpretation of results. Another option is the Bogardus social distance (BSD) scale. A BSD scale measures participants’ affect (e.g., intimacy, hostility, and warmth) toward outgroup members. BSD scales also use 5- or 7-point response options. For example, using a hypothetical 5-point response option along with our example from above, “1” would reflect desiring no social distance from transgender women while “5” would indicate desiring maximum social distance. Like SD scales, there may be discrepancies between participants’ interpretations of the options between desiring “no social distance” versus “maximal distance” from transgender women. The key difference between the two is the semantics of each item: Likert scales employ statements related to the construct of interest while BSD scales more commonly rely upon questions. 

### 4.3. Testing Content Validity

Consider our previous example involving the development of a measure of attitudes toward two-mother parenting. Imagine that you have now created a pool of 100 items. You have reviewed this pool and are satisfied with the quality of the items. They *seem* to be measuring the construct of interest; namely, attitudes toward two-mother parenting. However, your faith in the caliber of these items is insufficient. They must be evaluated formally.

Given the number of items that you have created, we recommend that you target four to five content experts. This number will ensure that experts are providing granular assessments of approximately 15 to 20 items each. You want to avoid tasking content experts with reviewing a full pool of items. Why? First, because it is unlikely they will do a thorough job (i.e., the experts may be more critical of the earlier items, and more “lenient” with the later items). Second, there is a risk that content experts might begin their review and then stop because they do not have the time required to assess 100+ questions. Third, if the review process is too labor-intensive, it might be difficult to recruit content experts (i.e., few will have the time needed to assess a pool of items containing 100+ items). 

Prior to approaching your content experts, it is critical that you meticulously proofread the items to ensure that your pool contains as few grammatical and spelling errors as possible. Having multiple “pairs of eyes” involved in this process is invaluable. 

The experts should be individuals with a background in psychometrics and/or expertise in the topic you are examining. In this hypothetical example, you would recruit experts who have published on the topic of same-sex parenting (ideally, two-mother parenting). A developmental psychologist, who is known for their parenting research, but who has limited experience with SGMPs, would *not* constitute a suitable expert. Each expert would review their subset of items on various dimensions including item clarity, item representativeness, and item quality. Given that assisting with this process is voluntary and time-consuming, we recommend that a small honorarium (e.g., a $10 gift card to a merchant of their choice) be provided. 

For each item, a mean rating would be calculated per dimension. Assume that, for item clarity, a 3-point scale was used: 1 = item is not clear; 2 = item needs revision to be clear; and 3 = item is clear. In this context, item clarity is subjective and based on the perception of the experts you approach. In addition to implementing a 3-point scale, you may also wish to request, time permitting, brief comments regarding *why* the experts believe specific items require revision (i.e., items receiving a score of 2) (see [35] for more details). Any item that has an average score < 2 would be removed from further consideration (i.e., an item regarded by experts as unclear would be eliminated). The outcome of this evaluative process will vary depending on the perceived quality of the items. For example, if most of the items are viewed as clear and representative, then—at the content validity stage—the pool will not have diminished appreciably (Fear not. We will discuss additional techniques that are useful in winnowing pools of items.). If, however, most of the items are perceived as unclear and non-representative of the construct of interest, then you will have to spend time revising and generating new items. The resultant pool then will need to be *reassessed* by content experts. Ideally, the individuals that evaluated the previous pool also will examine the revised one.

### 4.4. Quantitative Assessment of Item Integrity

Let us suppose that you have received feedback from content experts about your pool of items on same-sex parenting. A total of 50 of your 100 items were removed (i.e., 32 were viewed as unclear and 18 were seen as unrepresentative). An additional 15 items were seen as requiring revision. Given the size of your item pool, you elect to drop 10 of these items and revise the remaining 5. Thus, you now have a 40-item pool. You distribute these items to your content experts a second time. The feedback is positive (i.e., all items are seen as clear, representative, etc.). Does this mean you now are ready to distribute your 40-item measure to participants? Unfortunately, the answer is no. 

You still need to gauge whether any of the retained items are redundant; have limited variability (i.e., only one or two response options are selected by most participants); or are biased (i.e., responses to the items correlate strongly with responses on indices measuring social desirability). To assess such concerns, you need to distribute your item pool to a small sample of participants (e.g., 50 to 75 individuals). One caveat: this test sample should be like the type of sample you intend to use. Thus, in the case of our hypothetical same-sex parenting measure, if your goal is to distribute this scale to college/university students, then your test sample should also comprise post-secondary respondents. 

Participants would receive the test pool, which in this example is 40 items, and a psychometrically sound measure of social desirability bias (We recommend the Social Desirability Scale-17 [36].). A small number of demographic questions also should be included for diagnostic purposes. For example, you might find that scale score reliability coefficients are satisfactory for self-identified cisgender women, but not for cisgender men. Of course, your ability to conduct these sorts of tests will depend on the size of the test sample (e.g., if you only have five cisgender men, you will be unable to conduct any sort of psychometric testing with this group). 

After the data have been collected, you should compute frequencies for each item in the test pool. The goal is to remove items that possess insufficient variability. In our own scale development practice, we have relied upon [37]’s guidelines (We reiterate, again, that these are guidelines and not edicts.). First, you could remove any item in which more than 50 percent of responses fall into one response option. For instance, if 62% of respondents select “strongly agree” for question X, we recommend eliminating that item because it is providing insufficient variability (i.e., you are observing restriction of range, which has various statistical implications). Second, you could remove any item in which two combined response options total less than 10 percent. To illustrate, assume that 3% “strongly agree” and 5% “agree” with question X. That question should be eliminated because only 8% of participants agree with its content. Third, ref. [37] contends that the majority (i.e., >50%) of your response options should have a minimum endorsement rate of 10%. For instance, assume that you have reviewed the frequencies for the following item: “Two-mother parents are able to show warmth to their child/children”. You obtain the following values: 45% = strongly agree; 21% = agree; 3% = slightly agree; 9% = neither agree nor disagree; 8% = slightly disagree; 9% = disagree; 5% = strongly disagree. You might opt to remove this item from your pool because most of your response options (i.e., 5 out of 7) do not have a minimum endorsement rate of 10%. 

The application of [37]’s criteria should result in the removal of myriad items from your pool. Be warned: if the number of items removed is high, you may need to create additional items. You do not want a situation in which the pool of retained items is too small (i.e., <10). 

Let us assume that, from your initial pool of 40 items, 13 were removed following the use of [37]’s guidelines. You now have 27 items, which is close to your targeted length (i.e., 20 items); however, we will apply one additional assessment to remove any further items that are suboptimal. 

When computing Cronbach’s alpha coefficient, there is output that is helpful in eliminating items. We recommend focusing on two pieces of information. The first is called “Cronbach’s alpha if item deleted”. As you might expect, this tells you what your scale score reliability coefficient (i.e., Cronbach’s alpha) would be, if you removed the item in question from your scale. If the removal of an item does not produce a noticeable decline in Cronbach’s alpha, then the value of that item may be questioned. We recommend using a cut-off of 0.03. Specifically, if eliminating an item decreases Cronbach’s alpha coefficient by 0.03 (or greater), the item should be retained. However, if the decrease is <0.03, the item is a candidate for removal. Should the removal of an item substantially *increase* Cronbach’s alpha coefficient, this is a sound indicator that the item should be eliminated. 

The second piece of output that is useful is called “corrected item–total correlation”. This output refers to the correlation between responses to the item in question and the sum of all remaining items. So, a corrected item–total correlation of 0.52 for item 1 details the correlation between responses on item 1 and the sum of all other items (e.g., items 2 through 27 in the hypothetical attitudes toward same-sex mothering instrument). You want all corrected item–total correlations to be positive. Negative correlations suggest that *higher* scores on a given item are associated with *lower* total scores on all remaining items, a result that, assuming all items are scored in the same direction, does not make conceptual sense. Additionally, based on our experience, we recommend that correlations fall between 0.40 and 0.60. Lower correlations (i.e., <0.40) suggest that the item may not link conceptually with the other items; higher correlations (i.e., >0.60) reveal possible redundancy (i.e., you have multiple items that might be assessing the same sliver of content). To summarize, items that correlate negatively with item–total scores or have correlation coefficients < 0.40 or >0.60 are candidates for removal. 

Another strategy that can be used to identify and remove weak items involves correlating the scores for each item with the total score on a measure of social desirability bias. In our own practice, we use a cut-off of 0.33 (i.e., any item that correlates at 0.33 or higher, suggesting approximately 10%+ shared variance, with a measure of social desirability bias should be removed). Given that pilot test samples are (typically) small, the magnitude of the correlation is more important than its statistical significance. 

The quantitative assessment of item integrity involves applying numeric guidelines to identify items that should be deleted from an item pool. However, you want to avoid *blind adherence* to these sorts of recommendations. Refining your pool of items is an intricate dance between maximizing the quality of the items, reducing the size of the item pool to a manageable number, and ensuring sufficient content representativeness. In practice, there may be times when items are retained because you think they assess important facets of the construct—even though they fall outside the benchmarks we have listed. Let common sense prevail: if you believe that an item is important, at this stage, keep it. Once you have refined your item pool, you will want to assess its factorial validity.

### 4.5. Testing Factorial Validity

Factorial validity is not laden with various subtypes. However, computing evidence regarding this form of validity is arguably the most complex. Adding to this complexity is that we have different statistical methods available to us that should follow a specific order. The first step in assessing factorial validity involves exploratory factor analysis (EFA). EFA allows us to identify how many factors our new measure includes and how well the items load on each factor. In other words, we are constructing a theory regarding how the items in our new measure can be organized. EFA is an intricate technique that requires researchers to make a series of well-informed decisions regarding how the data should be treated. Ill-informed decisions may lead to results that could question the utility of one’s new measure. With EFA, we must be cognizant about (1) sample size; (2) extraction methods; (3) rotation; and (4) extraction decisions.

As noted earlier, a general rule of thumb is that a minimum of 200 participants should be recruited when under “moderate conditions” [25]. In this case, “moderate conditions” merely refers to situations where three or more items load onto each factor and communalities fall between 0.4 and 0.7 (Communalities refer to how much one item correlates with all other items.). If you have recruited the number of participants suggested above, their data can be used for this analysis. 

Extraction methods fall under two distinct models: “common factor” (CF) and “principal component analysis” (PCA). A common knowledge gap regarding these models is that each serves a different purpose and cannot be applied interchangeably. In other words, only methods falling under the CF model can execute an EFA. For context, the PCA model is only suitable for situations where researchers are concerned with item reduction; it cannot and does not consider how individual items load onto certain factors (which is exactly what we want!). Unfortunately, the PCA model is regularly reported as the extraction method used to assess factorial validity, particularly among scales relevant to LGBTQ2S+ persons (e.g., see scales reviewed by [3,6,7,8,9,10]). This may be because it is the default setting in many common statistics packages (e.g., SPSS).

To reiterate, ensure that you choose methods rooted in the CF model, of which there are several. One recommendation is the maximum likelihood (ML) method. ML is an excellent choice because it is capable of handling data that moderately violate normality assumptions. For situations where one’s data severely violate normality assumptions, the principal axis factoring (PAF) method is recommended as an alternative strategy. While it may seem logical to just “always” use PAF “just in case”, there are certain benefits of ML over PAF. For example, ref.[38] note that ML can produce more robust goodness-of-fit indices compared to PAF. 

The term “rotation” refers to how your statistics program will situate the axes (i.e., each of your factors) to better coincide (i.e., “fit”) with your individual data points, effectively simplifying the data structure. There exist two main forms of rotation: orthogonal and oblique. Orthogonal rotation yields factors that are uncorrelated, which is problematic in the context of SGMPs since most constructs of interest will yield factors that are related to some degree. As a result, the use of orthogonal rotation will not help us determine simple structure. Oblique rotation, on the other hand, allows, but does not require, factors to be correlated. Given this enhanced flexibility, oblique rotation should always be utilized. Unfortunately, orthogonal rotation methods are often used, likely due to them being the default in commonly used statistical software (e.g., SPSS). There are several oblique rotation methods available (e.g., quartimin, promax, and direct oblimin) but none have emerged as a superior method [38]. Therefore, selection of any oblique rotation method should yield similar results. 

Finally, we need to decide how many factors we should extract based on our EFA. A common approach is to select all factors that possess eigenvalues > 1. However, this approach is completely arbitrary and often leads to overfactoring; hence, it is not a useful strategy. Parallel analysis (PA) is a data-driven method that has been identified as the most accurate (although still prone to occasional overfactoring). As a result, PA should be used in conjunction with a scree test (plotted eigenvalues) to serve as a “check-and-balance” [39]. While not an on-board function in common statistics programs (e.g., SPSS and SAS), ref. [40] maintains a syntax file that can be downloaded and easily used to run PA. Ultimately, this final step will help you determine whether your new scale possesses one or more factors. Items that do not load onto any factor or load onto more than one factor should be disregarded. If this process happens to lead to a situation where fewer than 10 items remain, more items may need to be created/added and previous steps carried out again with a new sample. 

Once we have selected our factors, it is a good idea to test the reproducibility of our factor structure. This can be achieved using confirmatory factor analysis (CFA). Completed via structural equation modeling, CFA should only be used when there is a strong underlying theory regarding the factor structure of our measure. If we consider our hypothetical attitudes toward same-sex mothers measure, EFA should precede CFA to allow for a robust theory regarding the factor structure we are aiming to confirm. It is important to note that CFA should be conducted with a different sample than was used for our EFA. Since CFA is more advanced than EFA, we recommend consulting other primers that exclusively focus on its various steps and interpretations of their outcomes (e.g., [41]). 

### 4.6. Testing Criterion-Related Validity

After completing the previous steps, you should be approaching your targeted number of scale items. Do not worry if your scale is still a bit too long (i.e., four to six items above the desired maximum). Criterion and construct validation processes will enable you to “fine-tune” your measure to reduce its length.

For criterion-related validity, we will focus on the concurrent form, in which you correlate scores on the measure you are creating with scores on a psychometrically sound (i.e., gold standard) measure designed to assess the *same* construct. As it is seldom used by attitudinal researchers, we will forgo discussing the other form (i.e., predictive validity). 

Provided a gold standard measure exists, testing concurrent validity is quite simple: you distribute your pool of items along with the gold standard indicator. Prior to summing your pool of items, you would assess scale score reliability. If the Cronbach’s alpha (or McDonald’s Omega) coefficient for your items is satisfactory, you would then correlate total scores on your items and total scores on the gold standard. 

If you have additional items from your pool that you would like to remove, an item-level analysis of concurrent validity may be performed. Here, you would inspect the correlation between each of the items you have created and the total score on the gold standard. A correlation coefficient of ≥0.33 is expected (i.e., you would anticipate that at least 10% of the variance in responses to each of your items could be accounted for by scores on the gold standard). Again, if an item from your pool has a lower correlation coefficient, it would suggest that this item does not have a strong conceptual association with the construct you are measuring. As a result, this item would be a candidate for removal.

### 4.7. Important Considerations about Construct Validity

If a gold standard indicator is available, you can test the concurrent validity of your item pool. However, as noted earlier, there may be instances where a gold standard does not exist. You may be testing a novel construct (i.e., one that has not been measured previously), or existing scales may be flawed in substantive ways and, consequently, unable to serve as a “gold standard”. It is important to note that, regardless of whether a gold standard exists, researchers *still* need to conduct tests of construct validity. Stated simply, you must demonstrate that scores on your proposed scale correlate, for theoretical and/or empirical reasons, with scores on other scales. 

Before we provide specific examples of how to test for construct validity, a few points need to be considered. First, the scales you select to test for construct validity must be psychometrically sound. Creating makeshift indices to correlate with your item pool does *not* provide compelling evidence of construct validity (or, more specifically, convergent validity). Nor is it sensible to treat, as validation indices, measures that have substandard psychometric properties. You must ensure that the validation measures you select are the “best of the best”. It is important not to settle for the first scale you come across that evaluates the variable you are using to test for construct validity. Also, you should not adhere blindly to the choices made by other researchers. For example, just because a researcher used the Anti-Fat Attitudes Scale (AFAS) [28], which we critiqued earlier, as a validation measure, should not offer reassurance that the AFAS would be appropriate for you to use in your own psychometric work. Exercise due diligence: ensure that *all* measures used in tests of construct validity were created in accordance with best practice recommendations for scale development. 

Following a thorough review of the literature, one should *always* opt for validation measures that have received *multiple* assessments of scale score reliability and validity. This review process also increases the likelihood that researchers will use the most up-to-date versions of validation measures. For instance, assume that we have created a scale assessing gay men’s sense of belonging to the LGBTQ2S+ community. As one strand of construct validity or, more specifically, convergent validity, we hypothesize that gay men reporting a greater sense of belonging should also be more open (i.e., “out”) about their sexual identity. To assess “outness”, we select a scale developed by [42], entitled the Outness Inventory (OI). A thorough review of the literature, however, would reveal that [43] subsequently revised the OI (i.e., items were added to measure outness to other members of the LGBTQ2S+ community). Thus, ref. [43]’s revised version would be a more appropriate choice. 

Second, construct validity involves furnishing multiple strands of evidence in support of your pool of items. We recommend generating three to five hypotheses per study, with the confirmation of each prediction offering one strand of support for the scale’s construct validity. To provide compelling evidence of construct validity, most of the hypotheses that you test need to be confirmed. In the absence of this type of confirmation, the psychometric integrity of your new measure is indeterminable. 

To test multiple predictions, the researcher can use multiple samples with sufficient power (e.g., 200+ participants per prediction) or a supersized sample (800+ participants) which can be partitioned into subgroups, with each subgroup being used to test different hypotheses. 

### 4.8. Testing Convergent Validity

Testing convergent validity is very similar to testing concurrent validity. Recall that concurrent validity is assessed when your new scale is compared to a psychometrically sound gold standard measure of the *same* construct. In contrast, with convergent validity, you are evaluating whether the total score on your new scale correlates with total scores on other measures that, *for theoretical and/or empirical reasons*, should correlate. So, for instance, if one developed a measure of internalized homonegativity (i.e., sexual-marginalized persons’ internalization of the opprobrium directed toward them by sexual majority group members—see [44]), one might test this measure’s convergent validity using the following predictions: (1) scores on the measure of internalized homonegativity will correlate positively with scores on a measure of loneliness (i.e., as internalized homonegativity increases, so, too, will loneliness); and (2) scores on the measure of internalized homonegativity will correlate inversely with scores on a measure of self-esteem (i.e., as internalized homonegativity increases, self-esteem decreases). The confirmation of these hypotheses would provide two strands of evidence supporting the convergent validity of this measure of internalized homonegativity. 

### 4.9. Testing Discriminant (or Divergent) Validity

The process is identical to the one articulated for convergent validity. The only difference is: with discriminant (or divergent) validity, you are predicting—again for theoretical and/or empirical reasons—that a statistically non-significant (or, at least, statistically negligible) correlation will be observed between scores on the measure you are creating and scores on a psychometrically robust indicant of another construct (Determining whether a correlation is “negligible” is a subjective process. Recognizing that, in the social sciences, “everything correlates to some extent with everything else” [45] (p. 204), we recommend using a cut-off of *r* = 0.33 (i.e., shared variance of approximately 10% suggests that a correlation coefficient is “meaningful”).). To illustrate, researchers have found that personality indices of neuroticism and extroversion correlate weakly with internalized homonegativity (see [46]). Thus, if you were developing a new measure of internalized homonegativity, you might opt to evaluate discriminant (or divergent) validity by testing the same predictions as those offered by [46] (i.e., participants’ levels of neuroticism and extroversion should be weakly associated with their levels of internalized homonegativity). 

### 4.10. Testing Known-Group (Discriminative) Validity

For this form of construct validity, two or more groups are compared on the scale that is being tested psychometrically. Typically, gender comparisons are made (i.e., for theoretical and/or empirical reasons, one predicts that self-identified men and women will evidence different total scores). However, known-group validity is not restricted to demographic variables. To illustrate, ref. [47] created a measure examining gay men’s disturbances in normal sexual responding (Gay Men’s Sexual Difficulties Scale, GMSD). To assess the GMSD’s known-group validity, the researchers compared those “at low risk” for anxiety and those “at risk”. As predicted, gay men “at risk” for anxiety obtained higher scores on the GMSD than their “low risk” counterparts. A similar grouping process was used with a measure of depression (i.e., GMSD scores for those “at risk” were compared to those deemed to be at “low risk”, with the former reporting higher overall GMSD scores than the latter). 

## 5. Final Observations

By following the steps that we have outlined, you should now be left with a scale that falls between 10 and 20 items. These items should be representative of the specific construct that you intend to measure. There should be strands of evidence attesting to the reliability and validity of the scores obtained from your new scale which will highlight it as a cutting-edge advancement in SGMP research. However, a few final “housekeeping” issues remain. The first involves naming your scale. We recommend selecting a title that delineates the construct being measured yet also differentiates it from rival scales. For example, although the scales have different foci, ref. [13]’s Modern Homonegativity Scale has the same acronym as [48]’s Modern Homophobia Scale. Therefore, it is important to conduct a thorough review of the literature to ensure that the name you give your scale is unique. Selecting a name that forms an acronym which can be pronounced phonetically might be advantageous (Pay attention to the acronym for your scale. TGM recalls a student creating a measure that she entitled “Feelings about Thinness”. The student subsequently changed the title when informed that the resultant acronym was FAT.). While creating scale names with whimsical acronyms is by no means a requirement, it may be useful in drawing eyes to your work and encouraging other researchers to implement your measure. 

In terms of other researchers using your new measure, it is important to point out that some institutions have strict policies regarding the use of previously developed measures and insist on researchers contacting the original authors to solicit permission. If, like us, you are interested in contributing to the SGMP literature and wish to encourage widespread use of your new scale by any interested persons, we recommend including a permissions statement below your scale. A sample statement may read, “Researchers engaged in non-profit research and who have received ethical approval at their home institution or organization are welcome to use this scale. Please note that if such conditions are met, it is not necessary to contact the corresponding author to obtain permission”. This type of statement will reduce the amount of time you need to spend granting interested parties’ permission to use your measure.

Finally, some readers may notice that our discussion of exploratory factor analysis (EFA) is somewhat superficial. This is an intentional decision on our part. As mentioned, EFA is an intricate technique that requires a series of well-informed decisions. Therefore, we strongly advise researchers who do carry out an EFA to consult primers whose focus is on this statistical technique. Two that we have found to be particularly helpful are [38,49].

## 6. Conclusions

**Figure 1:**
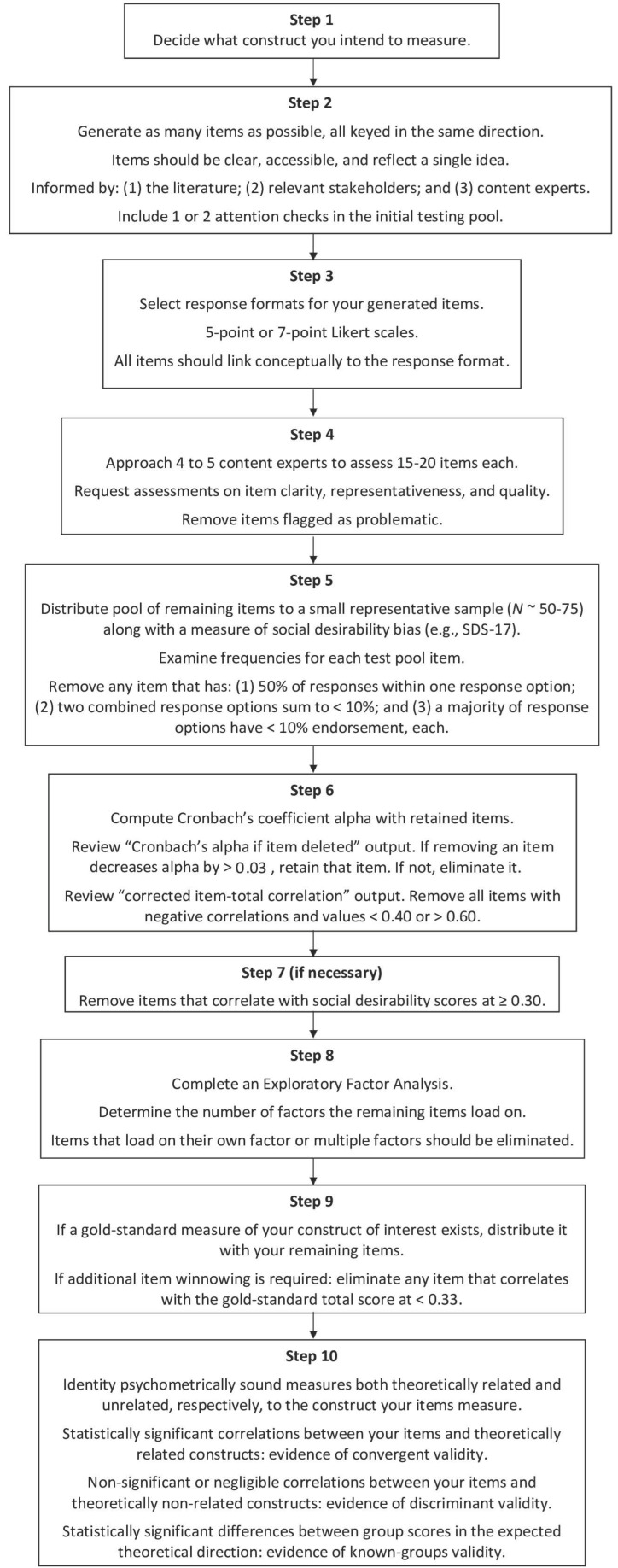
Summary of proposed steps to follow during the process of scale development. Note: these steps are intended as recommendations. There may be situations where some deviation is warranted.

Throughout this primer, we have sought to provide accessible step-by-step instructions intended to guide researchers through the process of scale development. Our recommendations, while rooted in best practices, are also informed by our various psychometric experiences (both positive and negative). We believe that the recommendations offered in this primer give a solid foundation for researchers who wish to develop measures of importance to sexual- and gender-marginalized populations. For convenience, we offer a flowchart (see Figure 1) that provides a pared-down version of the steps described throughout this primer. Think of this figure as a “recipe card”; it will guide you through each of the steps detailed above. However, as mentioned at various points, situations may arise where deviating from our proposed steps and cut-off points is warranted. Ultimately, each researcher must remain vigilant and make choices that will benefit the measurement of the construct in question (i.e., it may be beneficial to make choices outside of our recommended parameters on occasion). It is our hope that by addressing concerns with inconsistent operationalization and collating best practice recommendations into a single source, new psychometrically robust scales will become available that will enhance our ability to conduct SGMP research that possesses maximal basic and applied value. 

## Data Availability

Data are contained within the article.
